# Characterization and Antifungal Activity of Essential oil of *Cymbopogon citratus*: Special Emphasis on Preliminary Fungicidal Mechanisms against *Candida auris* and *Cryptococcus neoformans*

**DOI:** 10.1007/s00284-026-04862-w

**Published:** 2026-04-01

**Authors:** Lívia Gurgel do Amaral Valente Sá, Islay Lima Magalhães, Bruno Coêlho Cavalcanti, Álvaro Gabriel De Souza Araújo, Marcos Vinicius Queiroz Ambrósio Correia, Thaís Lima Ferreira, Daniel Sampaio Rodrigues, Vitória Pessoa de Farias Cabral, Lara Elloyse Almeida Moreira Gomes, Leilson Carvalho de Oliveira, João Batista de Andrade Neto, Sérgio Ruschi Bergamachi Silva, Manoel Odorico de Moraes, Cláudio Costa dos Santos, Hélio Vitoriano Nobre Júnior, Cecília Rocha da Silva

**Affiliations:** 1 Drug Research and Development Center (NPDM), Federal University of Ceará, Fortaleza, CE Brazil; 2 School of Pharmacy, Laboratory of Bioprospection in Antimicrobial Molecules (LABIMAN), Federal University of Ceará, Fortaleza, CE Brazil; 3 Department of Physiology and Pharmacology, Federal University of Ceará, Fortaleza, CE Brazil; 4 Department of Engineering and Technology, Federal Rural University of Semi-Arid, Mossoró, RN Brazil; 5 Christus University Center (UNICHRISTUS), Fortaleza, CE Brazil; 6 Brain Institute, Federal University of Rio Grande do Norte, Natal, RN Brazil

## Abstract

In 2022, the World Health Organization published a list highlighting *Candida auris* and *Cryptococcus neoformans* as among the priority pathogens in need of new therapeutic alternatives. The essential oil of *Cymbopogon citratus* (EOCC) is used in folk medicine for its various properties, including antimicrobial activity. However, there are few reports of its activity against these yeasts and the possible action mechanism. EOCC was characterized by gas chromatography combined with mass spectrometry. Antifungal activity was determined by broth microdilution against *C. parapsilosis* ATCC 22,019, *C. krusei* ATCC 6258, *C. auris* 01256P, fluconazole-resistant *C. albicans* and *C. neoformans* strains. The possible mechanism of action of EOCC against *C. auris* and *C. neoformans* was investigated by flow cytometry and the alkaline comet assay. EOCC contained as major chemical compounds β-pinene (4.50%), neral (32.80%), geraniol (8.13%) and geranial (41.29%). EOCC had minimum inhibitory concentration (MIC50) of 32 to 256 µg/mL against *Candida* spp. and 32 to 128 µg/mL against *C. neoformans*. The antifungal effects of EOCC may be related to the high presence of neral and geranial detected in its phytochemical composition. The mechanism of action appeared to be related to mitochondrial dysfunction, an increase in reactive oxygen species and damage to fungal DNA, leading to apoptosis-like cell death.

## Introduction

The increasing number of immunocompromised people and climate change have aggravated the problem of fungal infections [1, 2] These infections can be superficial or invasive, the latter being responsible for a high mortality rate of approximately 2.55 million deaths per year [3]. Invasive fungal infections can be caused by *Candida* and *Cryptococcus* species, as well as other fungi of medical importance [3, 4].

Furthermore, in 2022 the World Health Organization (WHO) published a list of fungal pathogens for which more surveillance, research and strategies to prevent the spread of resistant strains are needed. This list of critical pathogens includes *Candida albicans* and *C. auris* [5]. Invasive infections caused by *Candida* species are responsible for 482,000 deaths per year, mainly caused by *C. albicans*, *C. parapsilosis*, *C. tropicalis*, *C. krusei* and *C. glabrata* [3, 6]. In addition, *C. auris* is of particular concern as an emerging pathogen associated with hospital outbreaks due to its ease of transmission and the development of resistance to several classes of antifungal drugs [2, 6].

In addition to *Candida* species, another yeast on the WHO list of critical pathogens is *Cryptococcus neoformans* [5]. This yeast is often associated with meningitis in immunocompromised individuals, especially those with HIV, and causes about 181,000 deaths annually [7, 8]. Care must be taken in the treatment of cryptococcal meningitis, since the antifungal drugs used can have cytotoxic effects. This reinforces the need to search for more therapeutic alternatives [7].

Natural products are a rich source of bioactive compounds that can be used for therapeutic purposes. Among these, extracts of the medicinal plant *Cymbopogon citratus* (DC.) Stapf, popularly known as lemongrass, have been extensively studied for their applications in the pharmaceutical, food and cosmetic industries [9, 10]. The essential oil of *C. citratus* (EOCC) has various therapeutic properties, including anti-inflammatory, antioxidant, anthelmintic, anticancer [9, 10], antibacterial [11, 12] and antifungal activity, as well as against antimicrobial-resistant strains [13, 14].

Despite the numerous pharmacological activities attributed to EOCC, there are no reports in the literature on its mechanisms of action against *Candida* species, particularly *C. auris*, and *C. neoformans*. Therefore, the main objective of the present study was to chemically characterize (gas chromatography coupled to mass spectrometry) and preliminarily evaluate the mechanisms of action behind the antifungal potential of EOCC against *C. auris* and *C. neoformans* through standardized methodologies such as broth microdilution testing, flow cytometry analyses, and genotoxicity (alkaline comet assay).

## Materials and Methods

### Extraction of the Essential oil

The fresh leaves of *C. citratus* (DC.) Stapf used in this study were collected in the municipality of Baraúna in the state of Rio Grande do Norte (Northeast Region of Brazil), located at 5°08’42.5’S and 37°35’53.0’W. Extraction was carried out by the hydrodistillation method using a Clevenger apparatus. The total extraction time was approximately two hours after boiling. After separation of the hydrolate, the EOCC was treated with anhydrous sodium sulfate (Na_2_SO_4_) to remove residual water [15].

### Gas Chromatography Coupled to Mass Spectrometry (GC-MS)

GC-MS analyses were performed with an Agilent 8860-GC/5977B-MS instrument equipped with an HP-5MS fused silica capillary column (30 m × 0.25 mm ID, 0.25 μm film thickness) with helium at 1.0 mL min^− 1^ as carrier gas. The injector and detector temperatures were set at 250 °C. After holding at 40 °C for 1 min, the oven temperature was increased to 200 °C at 4 °C/min, then to 310 °C at 10 °C/min and held for 14 min. Mass spectra were recorded in a range of mass-to-charge ratios (m/z) between 40 and 550. The essential oils (1%, v/v, in hexane) and a series of n-alkanes were injected in the splitless mode with volumes of 1.0 µl. All injections were preceded by a blank sample injection (hexane) to ensure there was no carry-over [15].

### Microorganisms used

Three collection strains were used: *C. parapsilosis* ATCC 22,019; *C. krusei* ATCC 6258; and *C. auris* 01256P from CDC B11903. In addition, five clinical strains were used, including one fluconazole-resistant *C. albicans* and four strains of *C. neoformans*. The *C. albicans* strain was isolated from a human blood culture. The *C. neoformans* strains were isolated from human cerebrospinal fluid. They all belong to the mycotheque of the Laboratory for Bioprospecting of Antimicrobial Molecules of Federal University of Ceará (LABIMAN/UFC). The strains were seeded on potato agar supplemented with chloramphenicol. The *Candida* spp. strains were incubated at 35 °C for 24 h, while the *C. neoformans* strains were incubated at 35 °C for 72 h.

### Drugs Analyzed

EOCC was dissolved in dimethylsulfoxide (DMSO) at an initial concentration of 1024 µg/mL. Fluconazole (FLC) and amphotericin B (Ampho B), purchased from Sigma-Aldrich (USA), were also used. FLC was dissolved in sterile distilled water and Ampho B in DMSO, which was used in the tests at a final concentration of less than 2.5%.

### EOCC Antifungal Susceptibility Test against *Candida* spp. and *C. neoformans*

The broth microdilution test was performed in polystyrene microplates with 96 wells according to the Clinical and Laboratory Standards Institute protocol M27-A3 [16]. RPMI 1640 culture medium (pH 7.0 ± 0.1) buffered with 0.165 M morpholinopropanesulfonic acid (MOPS) (Sigma, USA) was used. After culturing the *Candida* spp. strains for 24 h and the *C. neoformans* strains for 72 h, initial inocula were prepared at 0.5 on the McFarland scale. These inocula were then diluted in RPMI 1640 medium to give final inocula with concentrations of 0.5 to 2.5 × 10³ CFU/mL, which were added to the 96-well microplate.

EOCC was tested in a concentration range between 1024 and 2 µg/mL, while FLC was evaluated in a range between 64 and 0.125 µg/mL. Finally, the microplates were incubated at 35 °C (± 2 °C) for 24 h and 72 h for the *Candida* spp. and *C. neoformans* tests, respectively. The minimum inhibitory concentration (MIC) was determined by visual reading and defined as the lowest concentration capable of reducing fungal growth by 50% compared to the growth control (MIC50) [16]. *Candida* spp. strains with MIC ≥ 8 µg/mL for FLC were considered resistant, except for *C. auris* 01256P, for which there is no defined cutoff point [17].

### Flow Cytometry Procedures

Two representative strains were used to evaluate the possible mechanism of action of EOCC: *C. auris* 01256P and *C. neoformans* 1. Both were tested at concentrations of ½ x MIC50 (16 µg/mL) and MIC50 (32 µg/mL) of EOCC, in addition to Ampho B (4 µg/mL) as a mortality control. Untreated fungal cells were used as a negative control.

#### Preparation of Fungal Cells

Both *C. auris* and *C. neoformans* were incubated on potato agar plus chloramphenicol at 35 °C for 24 h and 72 h, respectively. Suspensions of *C. auris* were then prepared in 3 mL of yeast nitrogen dextrose (YND). *C. neoformans* was suspended in 3 mL of RPMI 1640. Both strains were then incubated again under the same conditions as described above. Finally, the treatments were added to the inoculum (10^6^ cells/mL) in RPMI 1640 medium (pH 7.0 ± 0.1) buffered with 0.165 M MOPS [18, 19].

After the strains were incubated for 24 h with the treatments, the cells were analyzed by FACSCalibur flow cytometer (Becton Dickinson, San Jose, CA, USA) to determine cell viability, mitochondrial transmembrane potential, production of reactive oxygen species (ROS) and phosphatidylserine externalization by staining with Annexin V. The tests were performed in triplicate, with 10,000 events evaluated and cell debris omitted.

#### Analysis of Cell Viability

The propidium iodide (PI; 2 µg/mL) exclusion assay was used to identify dead or dying cells after treatment, given that PI only penetrates and stains DNA in cells with compromised cell membrane integrity [20].

#### Analysis of Mitochondrial Transmembrane Potential

The rhodamine 123 marker (1 µg/mL) was used to assess the influence of test samples on mitochondrial transmembrane potential, as described by Silva et al. [20] and Neto et al. [21].

#### Analysis of ROS Production

Fungal cells were incubated with 20 µM CM-H2DCFDA [5-(e-6)-chloromethyl-2’,7’-dichlorodihydrofluorescein diacetate acetyl ester] for 30 min at 35 °C under light protection. They were then collected, washed, resuspended in phosphate buffered saline (PBS) and immediately analyzed [20, 21].

#### Annexin V labeling of Fungal Cells for Detecting Apoptosis-like cell Death

Fungal cells were incubated for 20 min in Annexin binding buffer containing 5 µl/mL of FITC Annexin V and 5 µl of PI from BD Pharmingen™ FITC Annexin V Apoptosis Detection Kit I (San Jose, USA) [20, 21].

### Assessment of DNA Damage using the Alkaline Comet Assay

The assays were performed as described by Silva et al. [19] and Silva et al. [22], with minor modifications. First, the fungal cells were subjected to the treatment described in Sect.  2.6.1. Next, the cells were centrifuged in an Eppendorf microcentrifuge for 5 min. They were then immediately washed with distilled water and resuspended in buffer S (1 M sorbitol, 25 mM KH₂PO₄, pH 6.5). Then, 20 µL of cell suspension (~ 10^6^ cells/mL) was dissolved in 0.75% low-melting point agarose containing 2 mg/ml zymolyase 20T (Seikagaku Corp., Japan), and immediately spread onto a glass microscope slide pre-coated with a layer of 1% normal melting point agarose. Agarose was allowed to set at 4 °C for 5 min. Slides were then incubated in ice-cold lysis solution (2.5 M NaCl, 10 mM Tris, 100 mM EDTA, 1% Triton X-100, and 10% DMSO, pH 10.0) at 4 °C for at least 1 h in order to remove cell membranes, leaving DNA as “nucleoids”. After, slides were placed in a horizontal electrophoresis unit and incubated with fresh buffer solution (300 mM NaOH, 1 mM EDTA, pH 13.0) at 4 °C for 20 min in order to allow DNA unwinding and the expression of alkali-labile sites. Electrophoresis was conducted for 20 min at 25 V (94 V/cm). After electrophoresis, the slides were neutralized (0.4 M Tris, pH 7.5), stained with ethidium bromide (20 µg/mL) and analyzed using a fluorescence microscope. All the above steps were performed under yellow light or in the dark in order to prevent additional DNA damage. Images of 100 randomly selected cells (50 cells from each of two replicate slides) were analyzed for each concentration of test substance. Cells were scored visually and assigned to one of five classes, according to tail size (from undamaged-0, to maximally damaged-4), and a damage index (DI) value was calculated for each sample of cells. DI thus ranged from 0 (completely undamaged: 100 cells x 0) to 400 (with maximum damage: 100 cells x 4).

### Statistical Analysis

The EOCC sensitivity assay was performed in triplicate and MIC50 values were obtained by the arithmetic mean. For flow cytometry and alkaline comet assays, data are presented as means ± S.E.M. and compared by analysis of one-way of variance (ANOVA) followed by the Newman-Keuls test (*p* < 0.05).

## Results

### Characterization of the EOCC

**Fig. 1:**
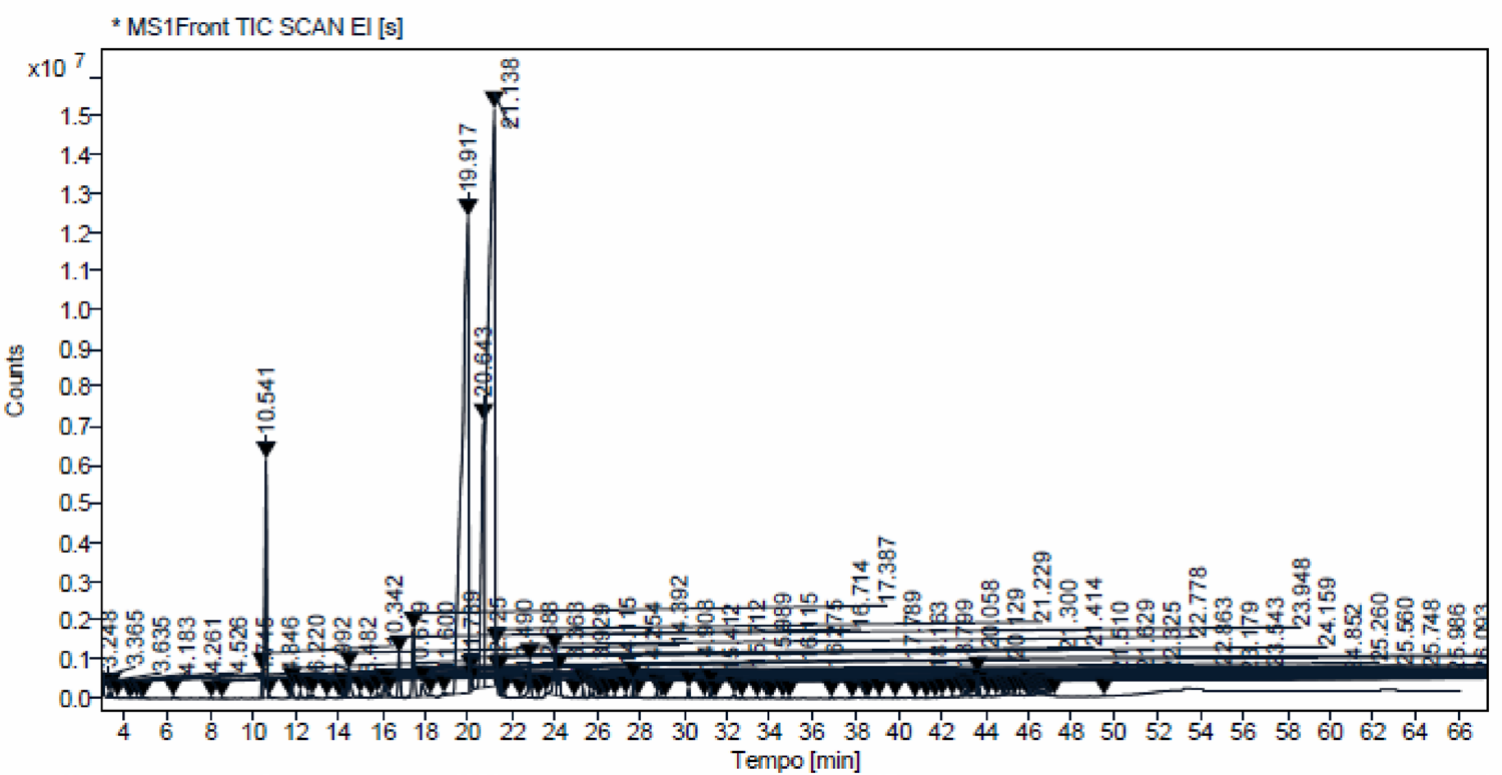
Chromatogram of EOCC obtained by GC-MS

**Fig. 2:**
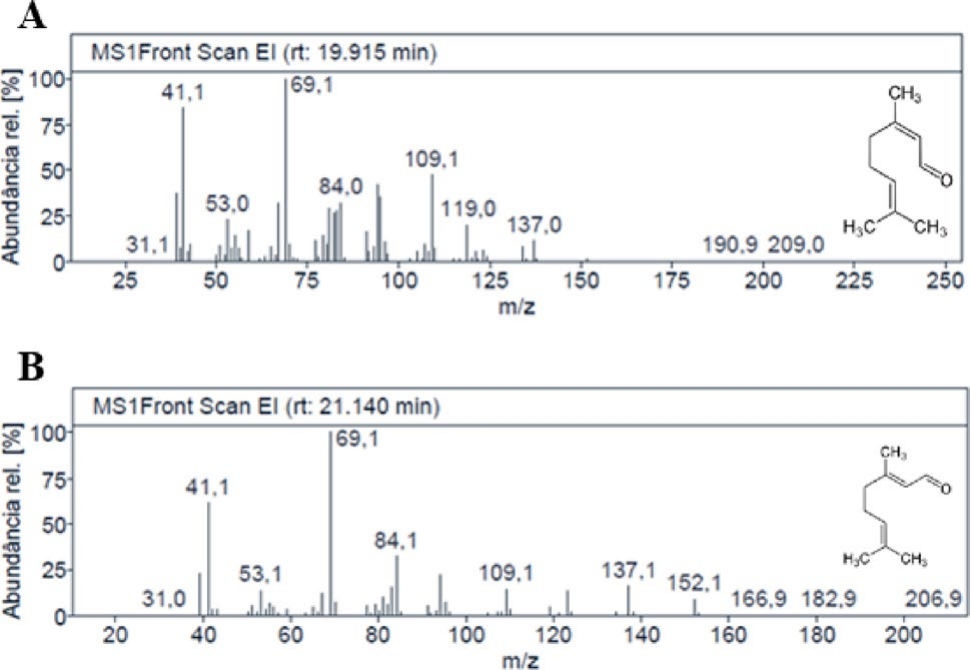
Mass spectrum and chemical structure of neral (A) and geranial (B) obtained from EOCC

Extraction of essential oil from fresh leaves of *C. citratus* by hydrodistillation yielded 6.7 mL/kg of plant material. It had a pale yellow color and a characteristic citrus odor. The chromatogram resulting from the GC-MS of the EOCC (Fig. 1) showed 33 chromatographic bands related to the constituents bioproduced from the leaves of *C. citratus.* The chemical composition was characterized by critical analysis of the spectra with retention time correction using the Kovats index [23] and subsequent comparison with literature data. The analysis of the spectra allowed characterization of approximately 92.5% of the total chemical composition of the analyzed material. The chromatogram of the EOCC (Fig. 1) had four main signals. The most relevant signals in percentage terms, with increasing retention times, referred to β-pinene (4.50%), neral (32.80%), geraniol (8.13%) and geranial (41.29%). The mass spectra are shown in Fig. 2.

The chemical composition of the essential oil distilled from the fresh leaves of *C. citratus* is given in Table 1 below, with data from the literature for comparison.

**Table 1:** Chemical analysis by GC-MS of *Cymbopogon citratus* essential oil (EOCC) compared with literature data

Compounds	KI [23]	KI experimental		GC-MS (%)	A	B	C
1. unidentified	-	-		-			
2. β-Pinene	979	992		4.50			-
3. *p*-Cymene	1020	1024		T			-
4. Limonene	1029	1028		0.24			-
5. (Z)-β-Ocimene	1037	1039		0.14			-
6. (E)-β-Ocimene	1050	1046		0.10			-
7. unidentified	-	-		-			
8. 6,7-Epoxymyrcene	1092	1092		0.13			
9. Linalool	1096	1099		0.77	1.10		1.29
10. unidentified	-	-		-			
11. *exo*-Isocitral	1144	1147		0.22			
12. unidentified	-	-		-			
13. Citronellal	1153	1155		0.10			0.12
14. (Z)-Isocitral	1164	1167		0.98			
15. (E)-Isocitral	1180	1184		1.69			
16. unidentified	-	-		-			
**17. Neral**	**1238**	**1258**		**32.80**	**31.00**	**33.20**	**33.31**
18. unidentified	-	-		-			
**19. Geraniol**	**1252**	**1279**		**8.13**	**8.08**		**3.05**
**20. Geranial**	**1267**	**1292**		**41.29**	**44.74**	**42.30**	**39.53**
21. unidentified	-	-		-			
22. 2-Undecanone	1294	1300		0.25			0.53
23. unidentified	-	-		-			
24. unidentified	-	-		-			
25. Geranyl acetate	1381	1385		0.63	5.98	3.20	0.24
26. 2-tridecanone	1496	1494		0.23			
27. unidentified	-	-		-			
28. Caryophyllene oxide	1583	1570		0.16	1.07		
29. unidentified	-	-		-			
30. Cubenol	1646	1650		0.12			-
31–33. unidentified	-	-		-			
**Total**				**92.48**			

Letter A represents the study by Anaruma et al. [28]; letter B represents the study by Kizak et al. [29] and letter C represents the study by Andrade et al. [30]

### Antifungal Activity of EOCC against *Candida* spp. and *C. neoformans*

The MIC50 values of EOCC ranged from 256 to 32 µg/mL for the different *Candida* species and from 128 to 32 µg/mL for the *C. neoformans* strains, as shown in Table 2.

**Table 2:** Minimum inhibitory concentration of EOCC against *Candida* spp. and *Cryptococcus neoformans*

Strains	MIC50 ^a^	
	Cymbopogon citratus essential oil (EOCC)	Fluconazole (FLC)
*Candida parapsilosis* ATCC 22,019	64 µg/mL	1 µg/mL
*Candida krusei* ATCC 6258	32 µg/mL	16 µg/mL
*Candida auris* 01256P *	32 µg/mL	1 µg/mL
*Candida albicans*	256 µg/mL	16 µg/mL
*Cryptococcus neoformans* 1*	32 µg/mL	1 µg/mL
*Cryptococcus neoformans* 2	32 µg/mL	2 µg/mL
*Cryptococcus neoformans* 3	128 µg/mL	2 µg/mL
*Cryptococcus neoformans* 4	32 µg/mL	2 µg/mL

^a^ MIC50 - Minimum inhibitory concentration (concentration capable of inhibiting fungal growth by 50%) after incubation for 24 h for *Candida* spp. strains and 72 h for *Cryptococcus neoformans* strains. Strains with * were selected as representative for each genus for flow cytometry and alkaline comet assays

### Analysis of Antifungal Mode of Action

#### Cell Viability after EOCC Treatment

**Fig. 3:**
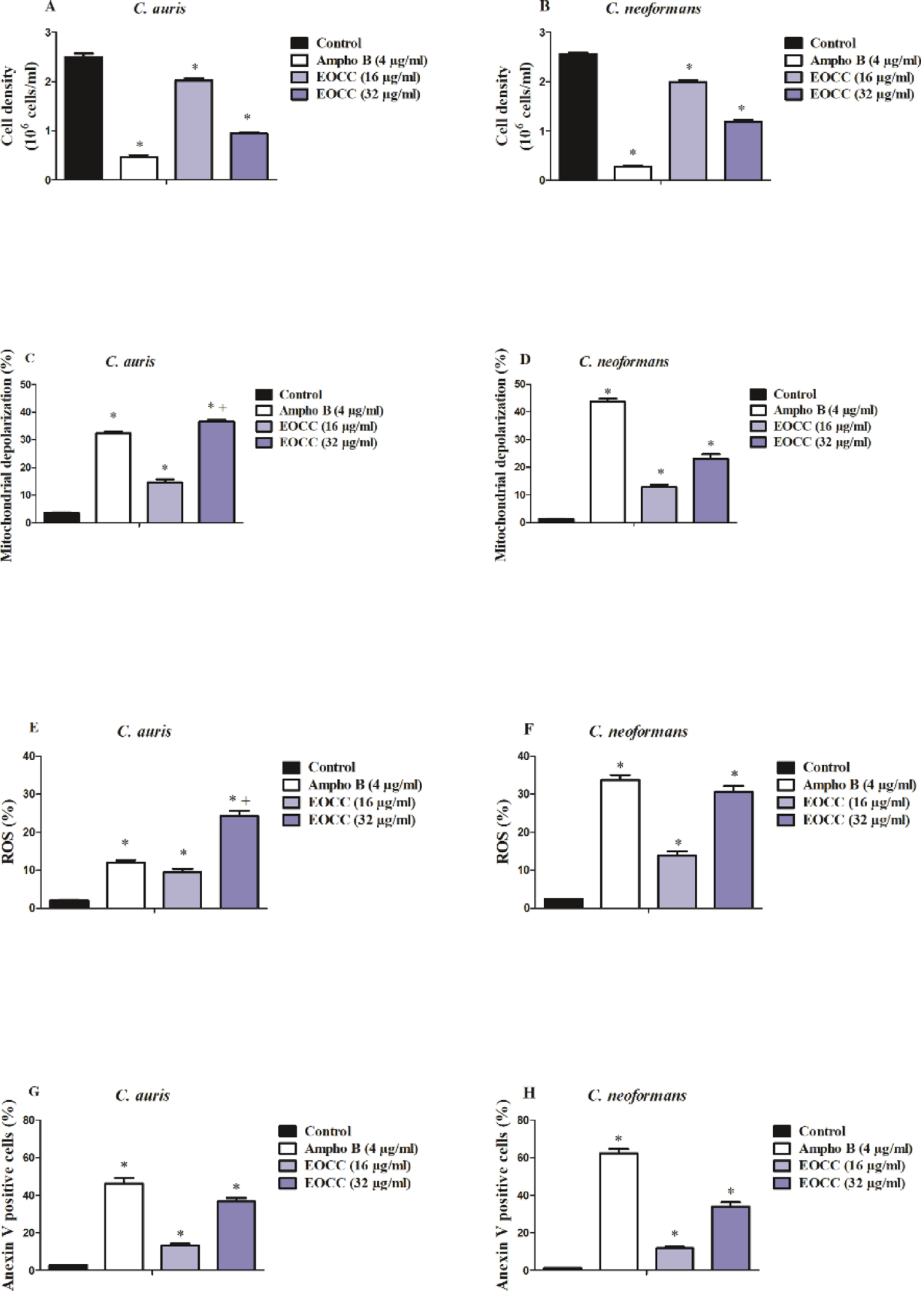
Possible antifungal mechanism of action of EOCC against *C. auris* and *C. neoformans* by flow cytometry. After treating *C. auris* and *C. neoformans* with the ½ x MIC50 (16 µg/mL) and MIC50 (32 µg/mL) concentrations of EOCC for 24 h, cell viability (A, B), mitochondrial depolarisation (C, D), ROS production (E, F) and Anexin V-labelled cells (G, H) of each species were evaluated, respectively. Ampho B was used as a death control and the negative control was untreated fungal cells of each species. * *p* < 0.05 compared to the control, submitted to ANOVA followed by the Newman-Keuls test

After 24 h of treatment with EOCC, both *C. auris* and *C. neoformans* cultures showed a similar reduction (*p* < 0.05) in their cell viability when compared to untreated controls. In *C. auris* cultures (Fig. 3A), EOCC induced a reduction of approximately 20 and 62% in the concentrations corresponding to the ½ x MIC50 and MIC50, respectively. For *C. neoformans*, EOCC exhibited an antifungal effect by reducing cell viability (*p* < 0.05) by around 22% and 54% for cultures exposed to the ½ x MIC50 and MIC50, in that order (Fig. 3B). Ampho B, used as a positive control, exerted a potent cytotoxic effect, reducing cell viability (*p* < 0.05) by more than 80% for both microorganisms.

#### Mitochondrial Depolarization Induced by EOCC Treatment

Treatment with EOCC was able to induce loss of mitochondrial membrane potential (*p* < 0.05) as demonstrated in Fig. 3. Evaluation of mitochondrial potential using the cationic dye rhodamine 123 showed that ½ x MIC50 and MIC50 of EOCC in *C. auris* resulted in a concentration-dependent decrease in mitochondrial potential in the range of 14.6% and 36.6%, respectively (Fig. 3C). Also, in *C. neoformans* cultured in the presence of EOCC, the loss of mitochondrial membrane potential was significantly observed for both concentrations evaluated: 12.8% (½ x MIC50) and 23% (MIC50). Ampho B, used as a control, significantly increased the percentage of cells with mitochondrial dysfunction (*p* < 0.05): 32.2% (*C. auris*) and 43.6% (*C. neoformans*).

#### Overproduction of ROS after Treatment with EOCC

*C. auris* cultures treated with ½ x MIC50 and MIC50 of EOCC showed a slight increase (*p* < 0.05) in intracellular free radical production compared to untreated cultures: 9.5% and 13.9%, respectively (Fig. 3E). ROS production was more evident in *C. neoformans* (Fig. 3F), where analysis of cells exposed to EOCC revealed an increase (*p* < 0.05) of 24.2% (½ x MIC50) and 30.6% (MIC50). Similarly, Ampho B induced a weak increase (*p* < 0.05) in ROS generation in *C. auris* (12.1%) and a more evident production in *C. neoformans* (33.7%).

#### Induced Apoptosis-like Cell Death after EOCC Treatment through Annexin V Staining

Cell death analysis via apoptotic mechanisms was monitored through Annexin V staining. Figure 3G, shows that after treatment period with EOCC, it was evident that a fraction of *C. auris* (13.2%; ½ x MIC50) were in the process of initial apoptosis-like cell death and with the increase in EOCC concentration (MIC50), the percentage of apoptotic cells more than doubled (36.7%). Interestingly, apoptotic cell fractions were similar in cultured *C. neoformans* (Fig. 3H), where ½ x MIC50 and MIC50 of EOCC induced a progressive increase in the order of 11.8% and 34%, respectively. As expected, Ampho B induced apoptosis-like cell death in 46.3% (*C. auris*) and 62.3% (*C. neoformans*) of treated cells.

#### DNA Damage in EOCC Treated Fungal Cells

**Fig. 4:**
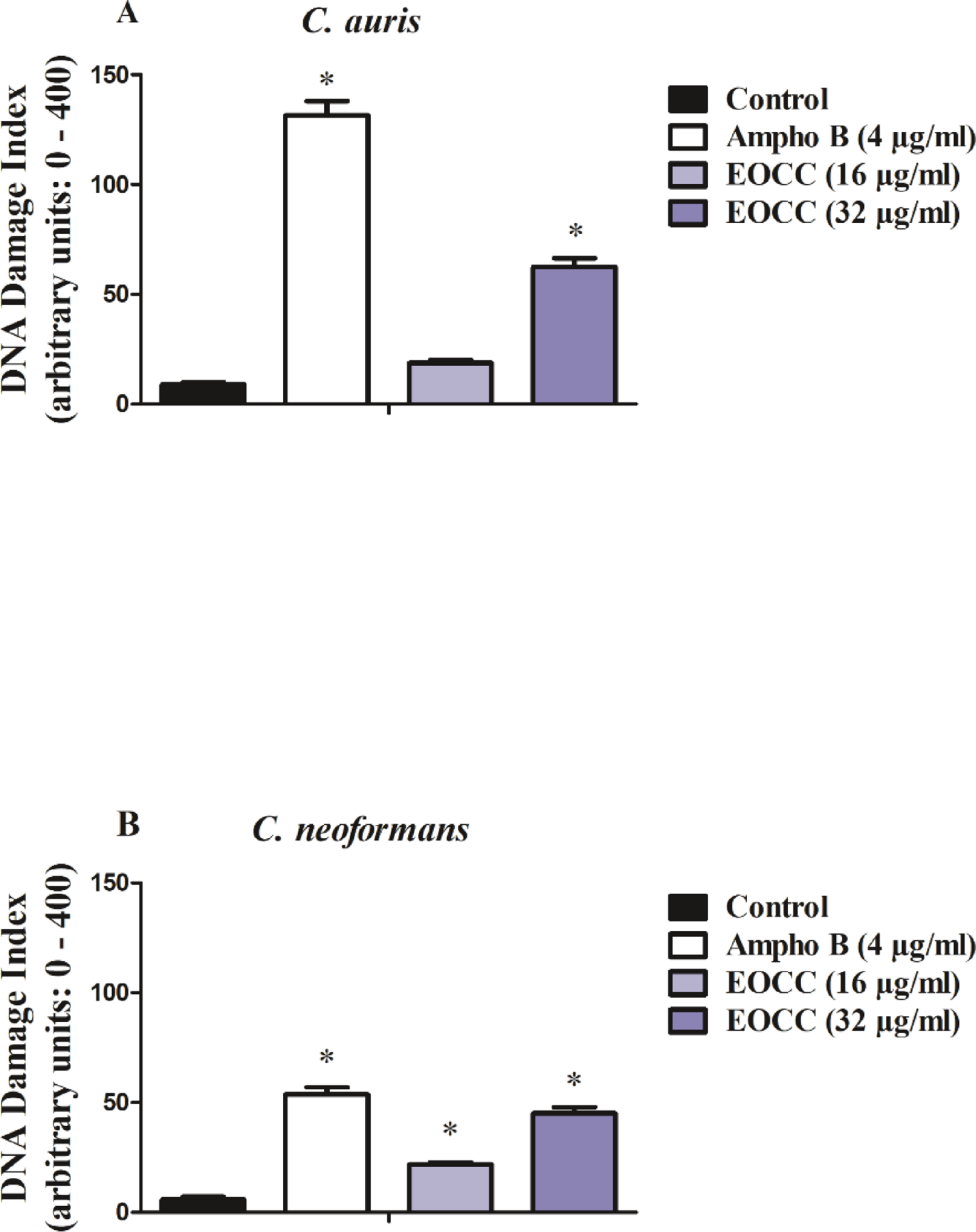
Alkaline comet assay of *C. auris* (A) and *C. neoformans* (B) cells exposed to EOCC. DNA damage caused by the treatments with the respective ½ x MIC50 (16 µg/mL) and MIC50 (32 µg/mL) values of EOCC of *C. auris* and *C. neoformans*, control (untreated cells) and Ampho B, as death control, after exposure for 24 h. * *p* < 0.05 compared to control, submitted to ANOVA followed by the Newman-Keuls test

Treatment of *C. auris* with EOCC induced a concentration-dependent increase (*p* < 0.05) in the number of fungal DNA strand breaks when compared to untreated cultures. The genotoxic effect was evaluated through the alkaline version of the comet assay by means of DNA damage index (DI) values. As shown in Fig. 4A, ½ x MIC50 of EOCC presented a low but significant DI value (18.83 ± 1.24; *p* < 0.05) compared to the control DI (9.00 ± 0.96) and *C. auris* treated with MIC50 of EOCC showed a DI value (62.5 ± 3.90) almost 7 times higher than the control. Furthermore, EOCC was also able to induce global lesions to the DNA of C. *neoformans*, since its concentrations related to ½ x MIC50 (DI 21.66 ± 0.98) and MIC50 (45.00 ± 2.96) resulted in an increase in DNA strand breaks higher (*p* < 0.05) than the basal DI values (5.81 ± 1.24) ​​of untreated cultures (Fig. 4B).

## Discussion

The bioactive compounds obtained from *C. citratus* have various applications in folk medicine, with the essential oil of this plant being the most widely used form [24]. The EOCC phytochemical analysis of current study revealed the presence of three main compounds, neral, geranial, and geraniol, with the first of two being basically the majority. Citral is an organic monoterpene formed by the mixture of the isomers neral and geranial and has antimicrobial properties against different species of *Candida* [25]. Citral has been reported to have antimicrobial properties against *C. parapsilosis*, *C. krusei*, and clinical strains of *C. auris* [26] and FLC-resistant *C. albicans* [27].

Our analysis of phytochemical composition of EOCC shows similarity with the chemical analyses of other *C. citratus* essential oils reported in the literature, with neral and geranial as the main compounds [28–30]. Herein, the non-selective activity of EOCC against various *Candida* species and *C. neoformans* was demonstrated by the different MIC50 values ​​shown in Table 2. Interestingly, among the *Candida* genus representatives used in this study, EOCC also showed activity against FLC-resistant strains (*C. albicans* and *C. krusei*).

The anticandidal properties of EOCC had already been reported against strains of *C. albicans* sensitive to FLC [14, 31]. In addition, the phytochemical composition of the essential oil [14] was similar to that of our study, also having neral and geranial as the two main compounds. To our knowledge, this may be one of the first studies to investigate the antifungal effects of EOCC on FLC-resistant strains of *C. albicans* and *C. auris*. This result is relevant in terms of the emergence of resistance to clinically used azoles in *C. albicans* and other non-*C. albicans* species, such as *C. auris*, which has recently emerged.

In addition, other studies have demonstrated the antifungal activity of EOCC against *Candida* species, with MIC50 values higher than those obtained in our study, ranging from 125 to 2,200 µg/mL [32–34] and from 0.8 to 3.9 µl/mL [35, 36]. In fact, the chemical constitution of essential oils is intrinsically related to their biological properties. Perhaps for this reason, Córdoba et al. [31] reported that EOCC had a greater antifungal effect on strains of *C. parapsilosis* ATCC 22,019 and *C. krusei* ATCC 6258 (with MIC equal to 50 and 1.6 µg/mL, respectively) in relation to our study, where the main constituent was myrcene (38.3%), which was not found in our analysis.

Furthermore, our microbiological analyses of EOCC also showed antifungal effects on the FLC-sensitive *C. neoformans*, which corroborates the studies by Khan [13], who reported that EOCC exerted antifungal activity against FLC-resistant strains of *C. neoformans* (MIC equal to 200 µg/mL). Khan [13] and Paula et al. [37] attributed the activity against *C. neoformans* to citral reported its effects against strains with different sensitivities to FLC, with MIC values ​​ranging from 7.8 to 1,600 µg/mL. These findings shed light on the potential use of EOCC in the therapy of cryptococcal meningitis, since some essential oils can cross the blood-brain barrier and thus act on the infectious agent [33, 18]. Thus, future studies should investigate whether EOCC can be a less cytotoxic and adequately effective therapeutic alternative for cryptococcal meningitis.

Emerging fungal pathogens pose significant threats to global public health. *C. auris* is an emerging health-associated pathogen of global concern. Over the past decade, its presence has been documented on all continents, highlighting its emergence as a highly transmissible and multidrug-resistant fungal pathogen [38, 39].

Regarding *C. neoformans*, this is a potentially fatal opportunistic pathogen that commonly affects the central nervous system of immunocompromised patients. Globally, this ringworm is responsib le for almost 20% of AIDS-related deaths [40]. Therefore, due to the growing concern about these pathogens in global health, we decided to preliminarily analyze the mechanisms of action associated with the antifungal properties exerted by EOCC in *C. auris* and *C. neoformans* cells. To our knowledge, this may be one of the first studies reported in the literature on the possible mechanism of action of EOCC against these two species.

Propidium iodide (PI) exclusion assay was used to distinguish live from dead cells. PI is a fluorescent dye that can not cross the intact plasma membrane of live cells but can enter and stain the DNA of dying or dead cells with damaged membranes. Analysis of our PI exclusion data corroborates the antifungal effects of EOCC, as it showed that the tested concentrations (½ x MIC50 and MIC50) significantly reduced the viability of the two pathogens evaluated here (Fig. 3A and B). Ngo-Mback et al. [33] showed that EOCC targets ergosterol in the plasma membrane of *Candida* spp. and this may also be the case of *C. neoformans* due to the high content of neral and geranial, which are the main compounds present in the EOCC analyzed here. This may have contributed to the reduction in cell viability seen in our results by affecting ergosterol biosynthesis.

Additionally, the two other most prevalent compounds in EOCC may contribute to reduced cell viability. Geraniol and β-pinene have been reported to interfere with ergosterol synthesis, and geraniol can inhibit PM-ATPase in *Candida* species, compromising cell membrane integrity [41, 42]. A molecular docking assay revealed that β-pinene has a high binding energy with delta-14-sterol reductase. This finding supports the hypothesis that β-pinene interferes with the ergosterol biosynthesis pathway. Furthermore, β-pinene was found to interact with 1,3-β-glucan synthase, which suggests interference with fungal cell wall biosynthesis [43].

The excessive formation of ROS and impairment of defensive antioxidant systems leads to a condition known as oxidative stress. The main source of free radicals responsible for oxidative stress is mitochondrial respiration. Moreover, EOCC was able to disrupt the mitochondrial potential of both test microorganisms, culminating in excess ROS production and the release of pro-apoptotic factors into the cytoplasm. EOCC-induced apoptosis-like cell death was detected by labeling with annexin V, which binds to phosphatidylserine exposed on the surface of apoptotic cells. Both *C. auris* and *C. neoformans* showed significant increases in the number of annexin V-labeled cells after EOCC treatment. Wani et al. [44] demonstrated that citral (mixture of the isomers neral and geranial present in EOCC as a major components) was able to inhibit antioxidant defenses, induce mitochondrial dysfunction, cell cycle arrest, and apoptosis-like cell death in *C. albicans*.

The deleterious effects of ROS on cellular biomolecules, including DNA, is a well-known phenomenon that can contribute to cellular damage and death. To evaluate the contribution of ROS on DNA damage in the fungi tested, we used the alkaline version of the comet assay that detects DNA damage, specifically DNA strand breaks and alkali-labile sites, in individual cells. We observed that EOCC increased the number of DNA lesions in both fungi evaluated here. This genotoxic effect may be linked to EOCC’s pro-oxidative potential.

Studies have already shown that citral (neral + geranial), a component of many essential oils, has been detected as the main constituent of EOCC, demonstrating that it possesses pro-oxidant properties (generation of ROS) against *C. albicans* [45]. Furthermore, the pro-oxidative effects of citral have also been described in bacteria, where Chueca et al. [46] showed that citral induces oxidative damage to the DNA of *Escherichia coli*. And also to better understand the biological activity of citral, Alam et al. [47] studied the in vitro mechanisms of its interaction with DNA using various biophysical techniques, concluding that citral is a DNA intercalating agent.

## Future Prospective

In addition to the findings presented in our study, it has previously been reported that EOCC can enhance the activity of conventional antifungal agents. In a study by Paiva et al. [32], EOCC was shown to enhance the activity of nystatin against *Candida* spp. strains. Furthermore, EOCC has been shown to inhibit the formation of biofilms by *C. tropicalis* and *C. albicans* on silicone surfaces and medical devices [36, 48]. This demonstrates its potential for coating medical devices, such as catheters, to prevent *Candida* biofilms from forming on them. This could be investigated further in future studies.

## Conclusion

The EOCC obtained from a region in Northeastern Brazil contained neral and geranial as its main chemical constituents. It also showed antifungal activity against FLC-sensitive and -resistant *Candida* strains, including *C. auris*, as well as *C. neoformans*. The mechanism of action of EOCC against *C. auris* and *C. neoformans* appears to be related to mitochondrial dysfunction, with increased production of ROS and damage to fungal DNA, leading to apoptosis-like cell death. Despite the promising results, further studies are needed to investigate the antifungal activity of EOCC to enable its use in clinical practice.

## Data Availability

Data sharing is not applicable to this article as no new data were created or analyzed in this study.
